# Supplementary material for: The Ecology of Nipah Virus in Bangladesh: A Nexus of Land-Use Change and Opportunistic Feeding Behavior in Bats
Source: Viruses. 2021 Jan 23;13(2):169. doi: 10.3390/v13020169 (PMC7910977; doi:10.3390/v13020169)
Supplement: Supplementary file 1 [file viruses-13-00169-s001.pdf]

Supplementary Materials: Supplementary tables and figures

The ecology of Nipah virus in Bangladesh: a nexus of land-use change and opportunistic feeding behavior in bats

Clifton D. McKee, Ausraful Islam, Stephen P. Luby, Henrik Salje, Peter J. Hudson, Raina K. Plowright, Emily S. Gurley

Table S1. Source information and distribution of spatial covariates across all mapped roost sites.

Covariate	Source	Timespan	Raster resolution	Median (IQR)
BIO1 – annual mean temperature (°C)	WorldClim [1]	1970–2000	1 km	25.4 (25.1–25.7)
BIO2 – mean diurnal temperature range (°C)				9.7 (9.4–10)
BIO5 – maximum temperature of warmest month (°C)				33.8 (33–34.6)
BIO6 – minimum temperature of coldest month (°C)				11.2 (10.8–11.6)
BIO12 – annual precipitation (mm)				1,937 (1,760–2,281)
BIO14 – precipitation of driest month (mm)				5 (3–8)
BIO15 – precipitation seasonality (coefficient of variation)				92.5 (86.2–98.5)
BIO18 – precipitation of warmest quarter (mm)				952 (689–1,148)
Distance to nearest artificial surface (km)	WorldPop/ESA-CCI-LC [2,3]	2011	100 m	8.1 (4.8–12.5)
Distance to nearest bare area (km)				28.9 (12.9–46.7)
Distance to nearest herbaceous area (km)				11.3 (5.5–20.7)
Distance to nearest shrub area (km)				50.8 (25.3–64.8)
Distance to nearest sparse vegetation area (km)				55.5 (32.7–81)
Distance to nearest tree area (km)				5.1 (2.2–9.3)
Distance to nearest inland water (km)	WorldPop/ESA-CCI-LC [2,3]	2000–2012	100 m	0.6 (0.3–1)
Distance to nearest waterway (km)	WorldPop/OSM [2,3]	2016	100 m	4.5 (1.3–8.5)
Distance to nearest road intersection (km)				4 (2.3–6.7)
Distance to nearest road (km)				1.3 (0.5–2.6)
Distance to protected wilderness (km)	WorldPop/IUCN [2,3]	2000–2017	100 m	197 (148–241)

Human population density (/sq km)	SEDAC/GPW [4]	2010	1 km	996 (858–1,260)
Elevation (m above sea level)	WorldPop [2,3]	2000	100 m	16 (12–24)
Slope	WorldPop [2,3]	2000	100 m	1 (0–1)
Night-time lights (VIIRS)	WorldPop [2,3]	2012	100 m	0.3 (0.2–0.5)
Forest pixels (>10% cover) within 15 km radius	Global Forest Change [5]	2000	30 m	60,151 (35,557–100,047)
Distance to nearest roost site (km)	This study	2011–2013	Points	1.5 (0.2–3.1)
Distance to nearest village (km)				1.8 (0.9–3.2)
Distance to nearest feeding site (km)				2 (0.9–3.6)
Distance to nearest date palm tree (km)				1.2 (0.2–2.7)
Roost sites within 15 km radius				7 (3–13)
Villages within 15 km radius				2 (1–4)
Feeding sites within 15 km radius				11 (3–20)
Date palm trees within 15 km radius				80 (29–307)

ESA-CCI-LC – European Space Agency Climate Change Initiative land cover, OSM – OpenStreetMap, IUCN – International Union for Conservation of Nature strict nature reserves and wilderness areas, GPW – Gridded Population of the World

Table S2. Selection of generalized linear models (GLM) for the number of districts affected by Nipah virus spillover based on selection by AICc. Only models with ΔAICc < 4 are shown.

**Model**	**DF**	**AICc**	**ΔAICc**
days_below17	2	85.219	0
days_below17 + DMI	3	87.867	2.648
days_below17 + MEI	3	88.024	2.805
days_below17 + SIOD	3	88.069	2.850
temp_mean + days_below17	3	88.126	2.907
temp_min + days_below17	3	88.127	2.908
days_below17 + precip	3	88.133	2.914

Table S3. Selection of generalized linear models (GLM) for the number of Nipah virus spillover events based on selection by AICc. Only models with ΔAICc < 4 are shown.

**Model**	**DF**	**AICc**	**ΔAICc**
days_below17	2	100.331	0
days_below17 + precip	3	102.867	2.537
days_below17 + DMI	3	102.914	2.583
temp_mean + days_below17	3	102.947	2.616
days_below17 + MEI	3	102.991	2.661
temp_min + days_below17	3	103.212	2.882
days_below17 + SIOD	3	103.243	2.912

Table S4. Sensitivity analysis for the association between the annual number of Nipah spillovers and the percentage of winter days below a temperature threshold, varying the threshold from 15 to 20 °C. The coefficient is the estimated coefficient for a Poisson GLM. All associations were statistically significant at the 0.01 level (**) or the 0.001 level (***).

**Outcome**	**Covariate**	**2001–2018**		**2007–2018**	
		**R^2**	**Coefficient**	**R^2**	**Coefficient**
Total_districts	days_below15	0.4	0.12***	0.82	0.13***
	days_below16	0.47	0.11***	0.83	0.11***
	days_below17	0.53	0.11***	0.7	0.09***
	days_below18	0.48	0.09***	0.6	0.07***
	days_below19	0.28	0.07***	0.45	0.06***
	days_below20	0.13	0.05**	0.3	0.06**
Total_events	days_below15	0.35	0.13***	0.78	0.14***
	days_below16	0.43	0.12***	0.85	0.13***
	days_below17	0.53	0.12***	0.79	0.11***
	days_below18	0.49	0.1***	0.72	0.09***
	days_below19	0.29	0.07***	0.55	0.08***
	days_below20	0.12	0.05***	0.33	0.07***

Table S5. Estimated coefficients for relationships between spatial covariates and bat roost occupancy (presence/absence of bats). Statistical significance of covariates based on estimated coefficients for the test data are shown as: not significant (NS) or significant at the 0.05 level (*), at the 0.01 level (**), or the 0.001 level (***).

Covariate	Lasso regression coefficient for training data (n = 380)	GLM coefficient for test data (n = 94)	GLM coefficient t-statistic
Intercept	0.69	0.95	3.8^{***}
BIO1 – annual mean temperature (°C)	0		
BIO2 – mean diurnal temperature range (°C)	0		
BIO5 – maximum temperature of warmest month (°C)	-0.028	-0.76	-1.5^{NS}
BIO6 – minimum temperature of coldest month (°C)	0		
BIO12 – annual precipitation (mm)	0		
BIO14 – precipitation of driest month (mm)	0		
BIO15 – precipitation seasonality (coefficient of variation)	0		
BIO18 – precipitation of warmest quarter (mm)	0.33	0.035	0.079^{NS}
Distance to nearest artificial surface (km)	0		
Distance to nearest bare area (km)	0.018	0.18	0.46^{NS}
Distance to nearest herbaceous area (km)	0		
Distance to nearest shrub area (km)	0		
Distance to nearest sparse vegetation area (km)	0		
Distance to nearest tree area (km)	0		
Distance to nearest inland water (km)	0.05	0.0092	0.026^{NS}
Distance to nearest waterway (km)	-0.17	0.2	0.65^{NS}

Distance to nearest road intersection (km)	0		
Distance to nearest road (km)	0		
Distance to protected wilderness (km)	0		
Human population density (/sq km)	0		
Elevation (m above sea level)	0		
Slope	0		
Night-time lights (VIIRS)	0		
Forest pixels (>10% cover) within 15 km radius	0		
Distance to nearest roost site (km)	0		
Distance to nearest village (km)	0		
Distance to nearest feeding site (km)	0		
Distance to nearest date palm tree (km)	0		
Roost sites within 15 km radius	0		
Villages within 15 km radius	0		
Feeding sites within 15 km radius	0		
Date palm trees within 15 km radius	-0.061	0.18	0.59^{NS}

Table S6. Estimated coefficients for relationships between spatial covariates and bat abundance (roost size). Statistical significance of covariates based on estimated coefficients for the test data are shown as: not significant (NS) or significant at the 0.05 level (*), at the 0.01 level (**), or the 0.001 level (***).

**Covariate**	**Lasso regression coefficient for training data (n = 255)**	**GLM coefficient for test data (n = 60)**	**GLM coefficient t-statistic**
Intercept	5.8	5.7	23.7^{***}
BIO1 – annual mean temperature (°C)			
BIO2 – mean diurnal temperature range (°C)			
BIO5 – maximum temperature of warmest month (°C)			
BIO6 – minimum temperature of coldest month (°C)			
BIO12 – annual precipitation (mm)			
BIO14 – precipitation of driest month (mm)			
BIO15 – precipitation seasonality (coefficient of variation)			
BIO18 – precipitation of warmest quarter (mm)	-0.2	-0.38	-1.9^{NS}
Distance to nearest artificial surface (km)			
Distance to nearest bare area (km)			
Distance to nearest herbaceous area (km)	0.076	0.33	1.4^{NS}
Distance to nearest shrub area (km)			
Distance to nearest sparse vegetation area (km)			
Distance to nearest tree area (km)			
Distance to nearest inland water (km)			
Distance to nearest waterway (km)	0.072	0.31	1.3^{NS}

Distance to nearest road intersection (km)			
Distance to nearest road (km)	-0.0046	0.05	0.19^{NS}
Distance to protected wilderness (km)	-0.058	-0.5	-1.7^{NS}
Human population density (/sq km)			
Elevation (m above sea level)			
Slope	0.055	-0.38	-1.4^{NS}
Night-time lights (VIIRS)			
Forest pixels (>10% cover) within 15 km radius	0.19	0.33	1.7^{NS}
Distance to nearest roost site (km)	0.038	-0.18	-0.87^{NS}
Distance to nearest village (km)	0.072	0.32	1.3^{NS}
Distance to nearest feeding site (km)			
Distance to nearest date palm tree (km)			
Roost sites within 15 km radius	-0.11	-0.11	-0.4^{NS}
Villages within 15 km radius			
Feeding sites within 15 km radius			
Date palm trees within 15 km radius			

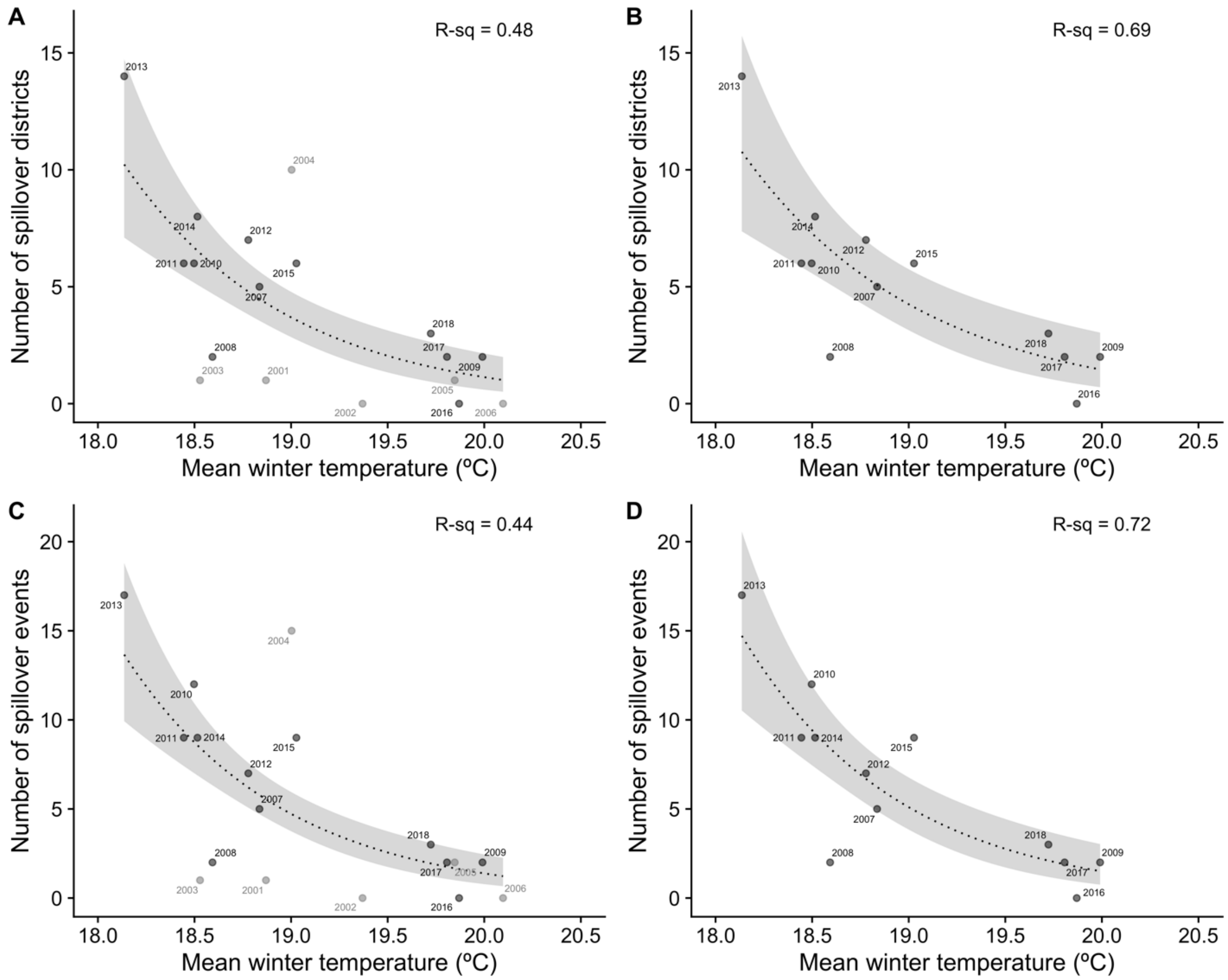

Figure S1. Variation in the number of Nipah spillover districts and events explained by mean winter temperatures. Panels show patterns for 2001–2018 (A, C) and 2007–2018 (B, D).

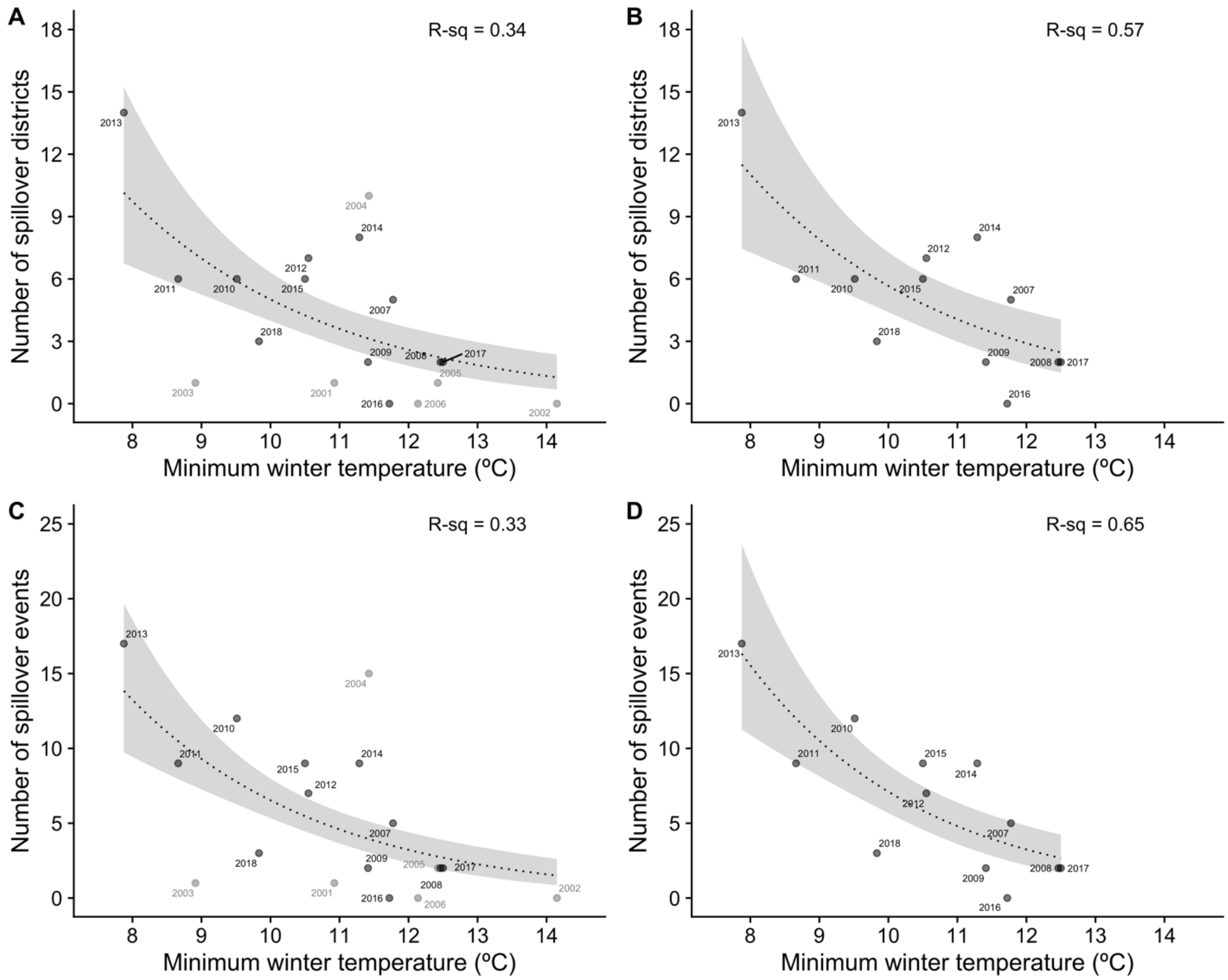

Figure S2. Variation in the number of Nipah spillover districts and events explained by minimum winter temperatures. Panels show patterns for 2001–2018 (A, C) and 2007–2018 (B, D).

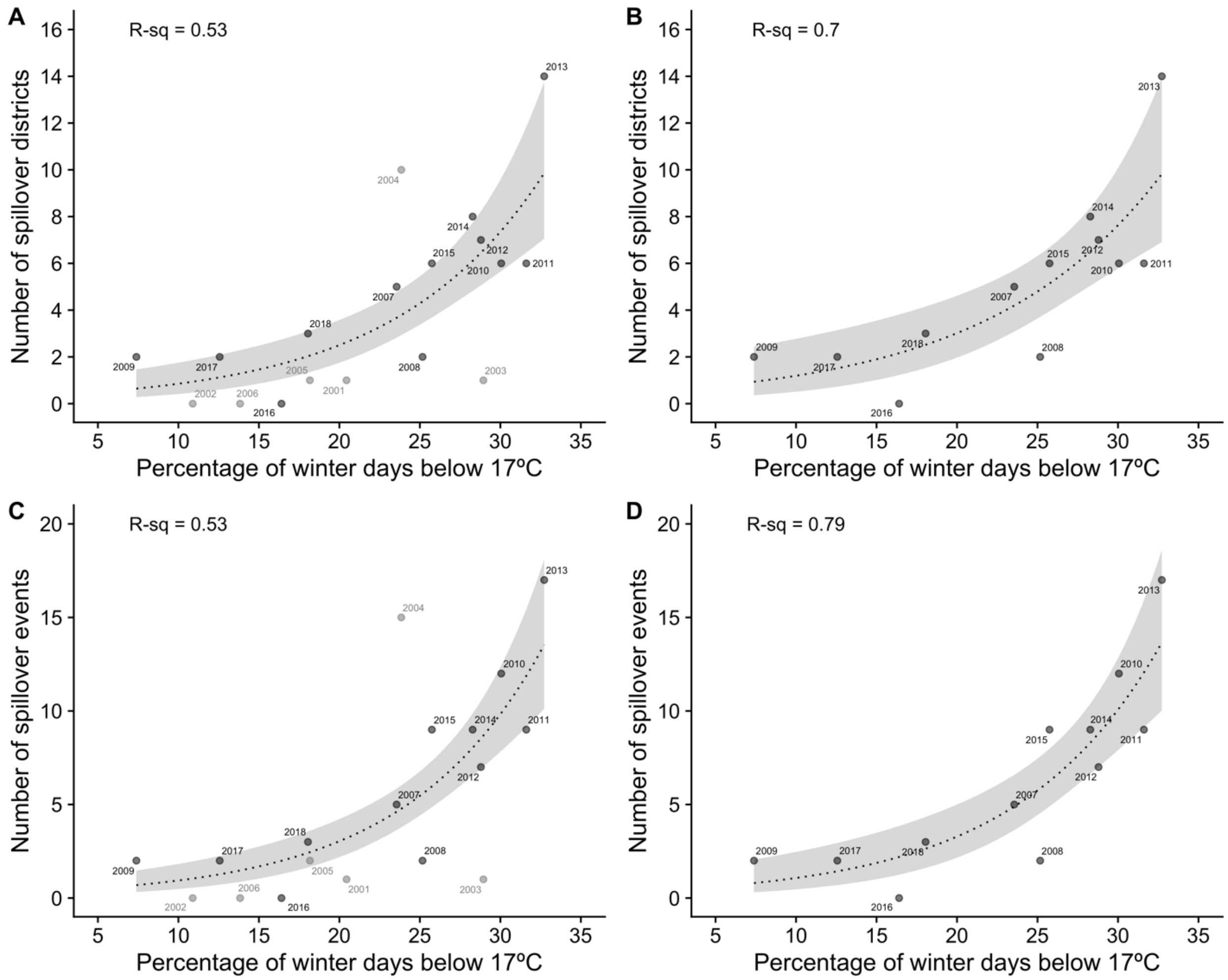

Figure S3. Variation in the number of Nipah spillover districts and events explained by cold winter temperatures. Panels show patterns for 2001–2018 (A, C) and 2007–2018 (B, D).

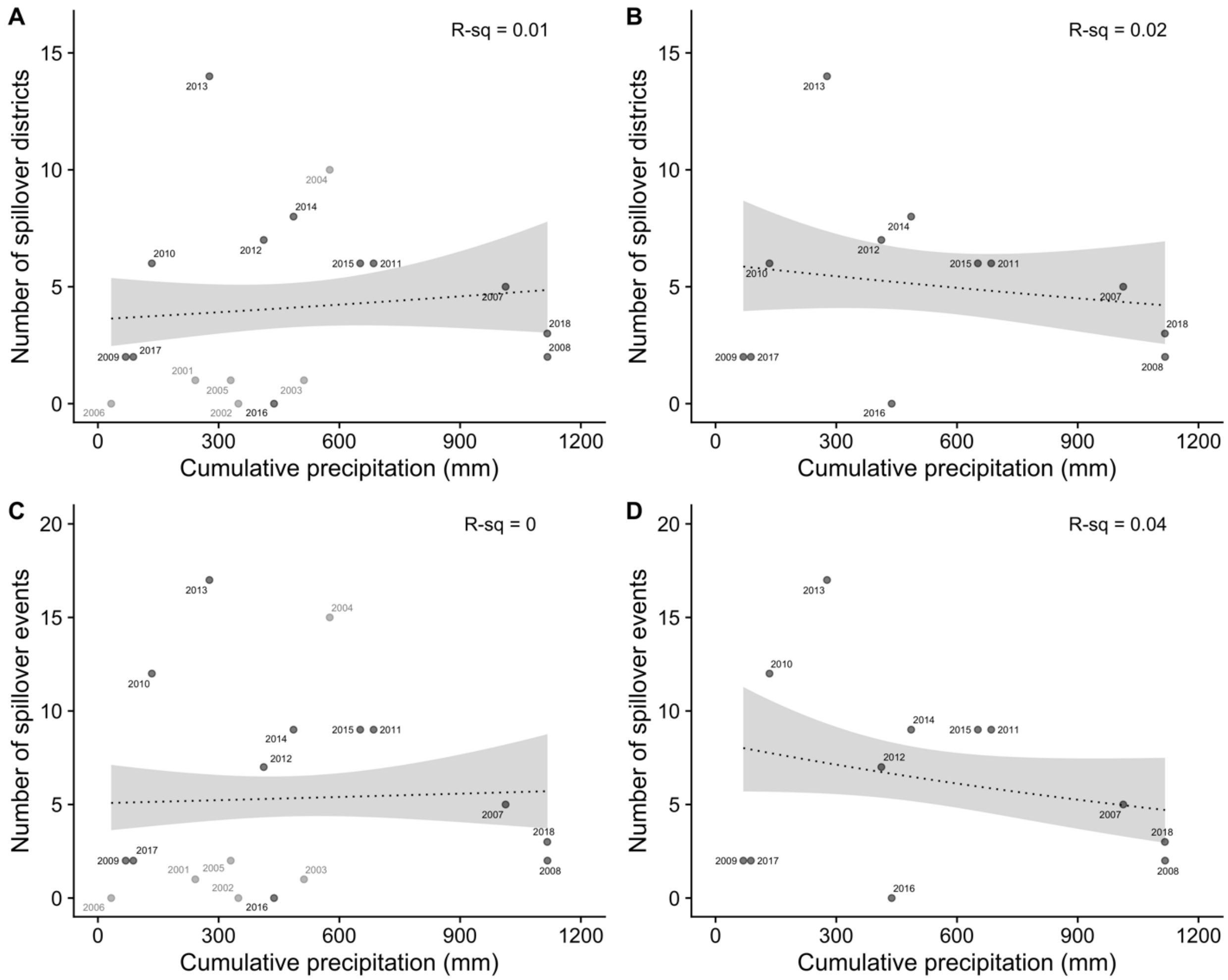

Figure S4. Variation in the number of Nipah spillover districts and events explained by cumulative winter precipitation. Panels show patterns for 2001–2018 (A, C) and 2007–2018 (B, D).

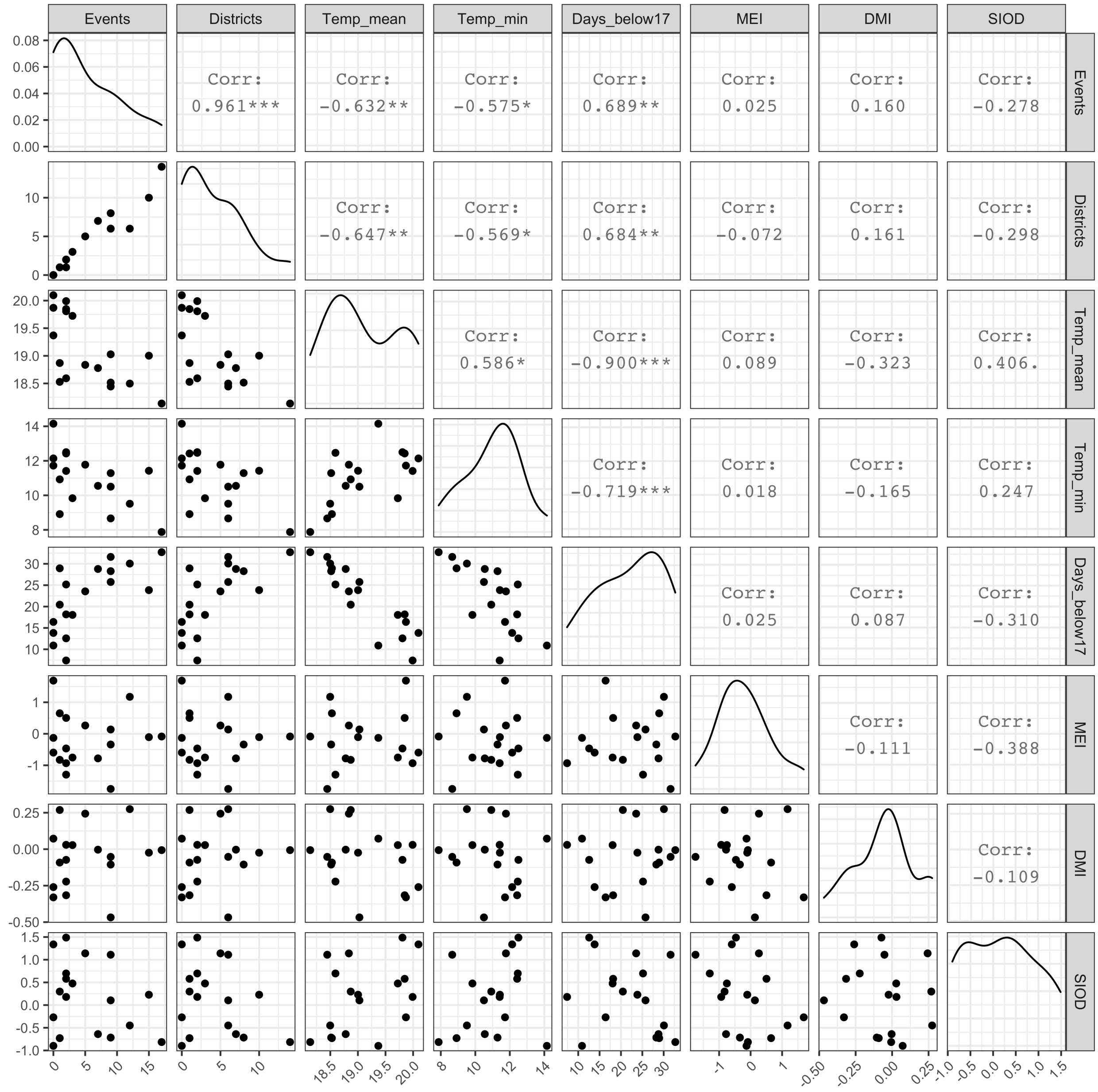

Figure S5. Pairwise Pearson's correlation between annual Nipah spillover events, spillover districts, and winter climate measures: mean temperature, minimum temperature, percentage of days below 17 °C, cumulative precipitation, and three indices of climate oscillations (MEI, DMI, and SIOD). Correlations with asterisks are statistically significant at the 0.05 level (*), the 0.01 level (**), and the 0.001 level (***).

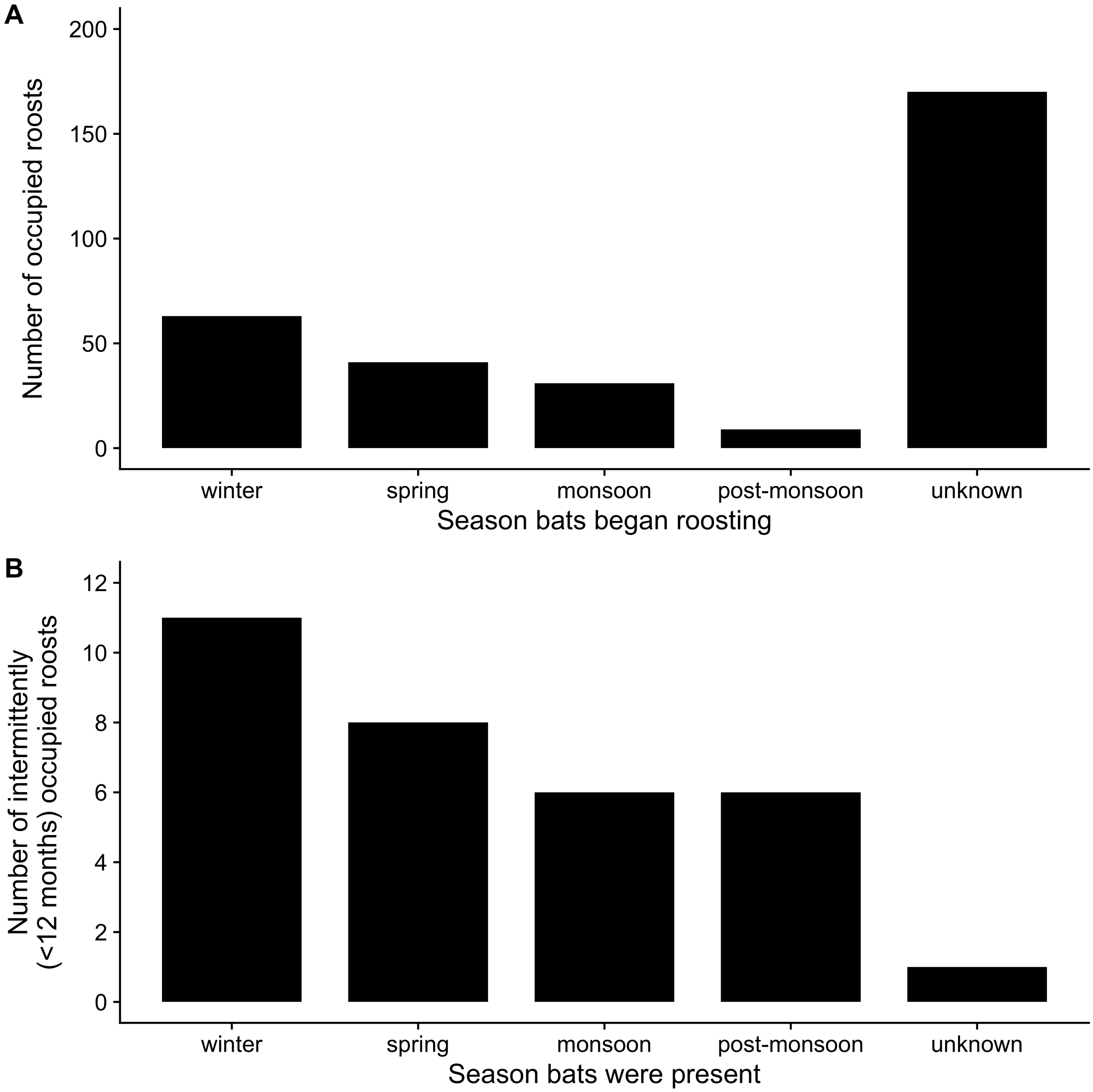

Figure S6. Seasonality of *Pteropus medius* roost site occupancy. Panel A shows the reported season when bats began roosting at the site. Panel B shows the season when bats were present at intermittently occupied roost sites (i.e., roosts were occupied <12 months of a year).

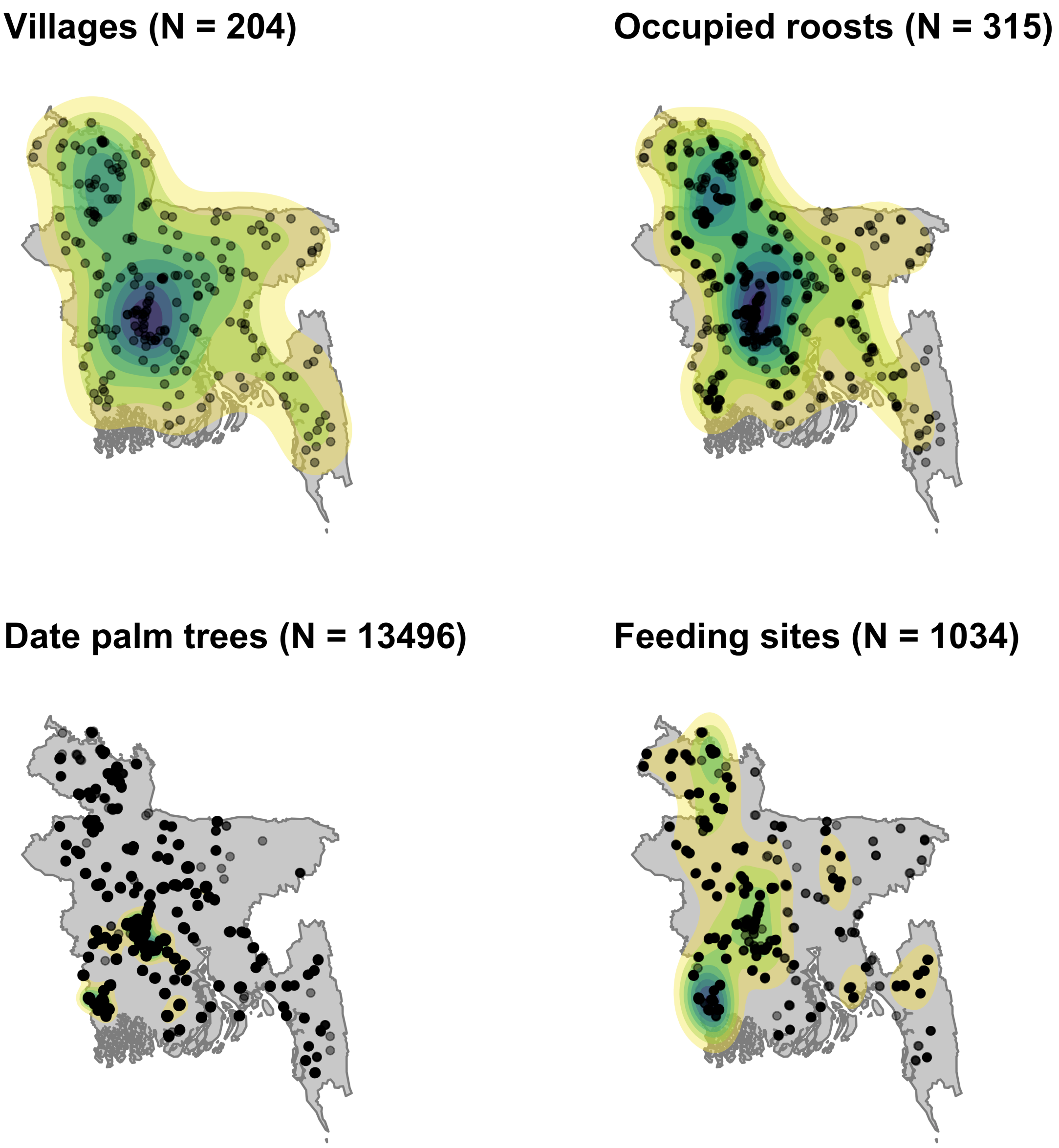

Figure S7. Spatial density of study villages, roosts, date palm trees, and bat feeding sites (fruit trees in and around villages). Color contours show the spatial density of events estimated with a bivariate normal kernel.

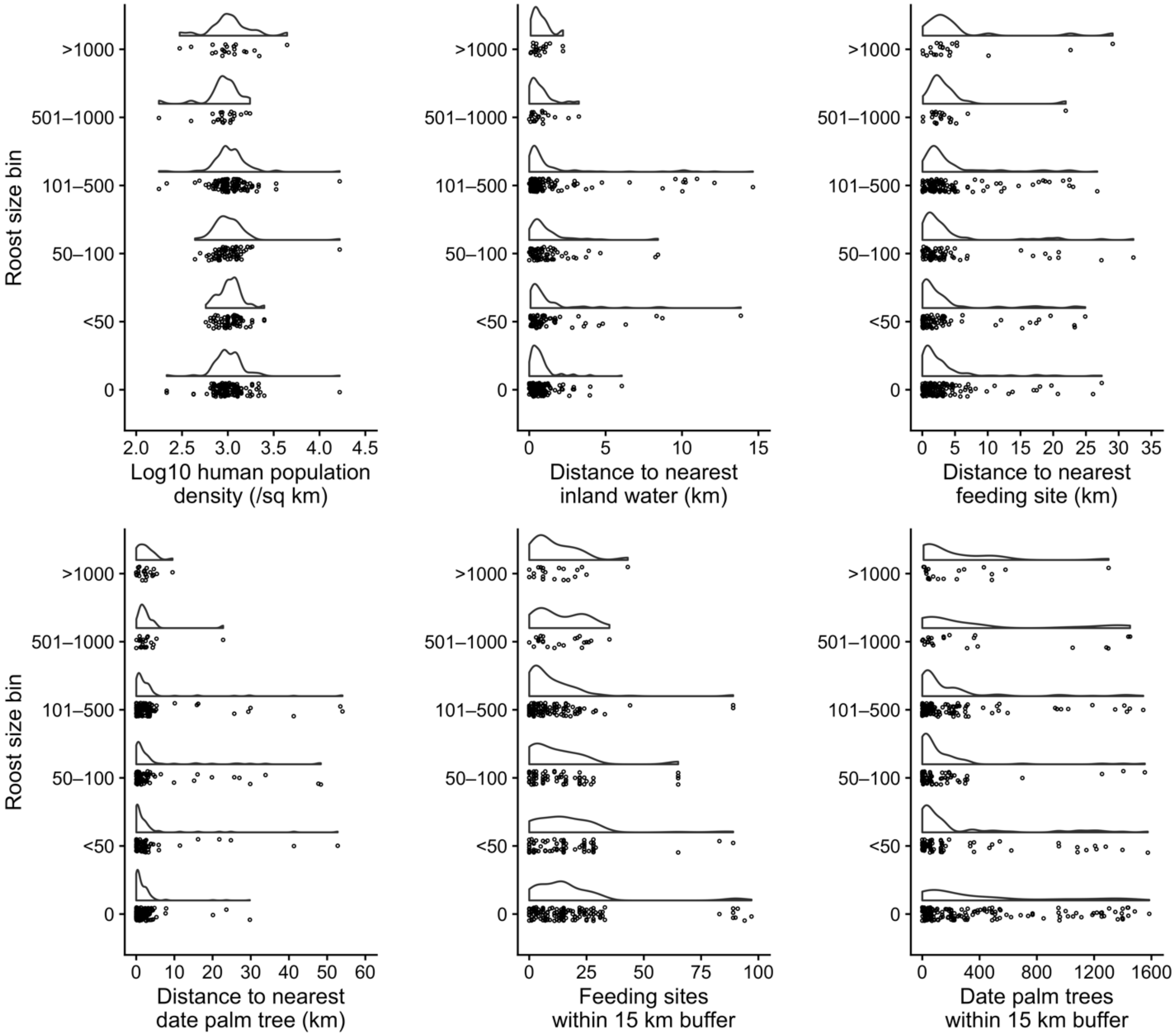

Figure S8. Distribution of roost sizes (including unoccupied roost sites) relative to select covariates in Table 2. Raincloud plots show the statistical distribution of variables over individual points.

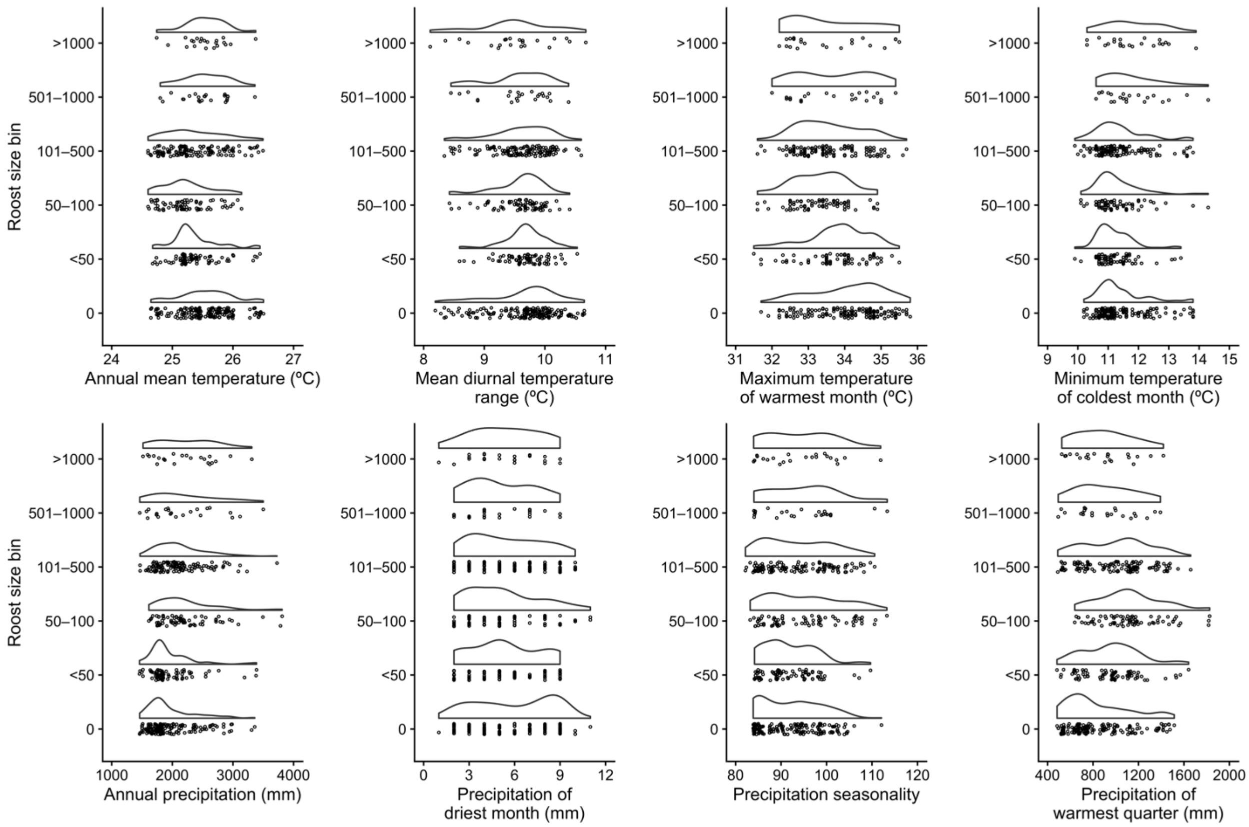

Figure S9. Distribution of roost sizes (including unoccupied roost sites) relative to bioclimatic covariates. Raincloud plots show the statistical distribution of variables over individual points.

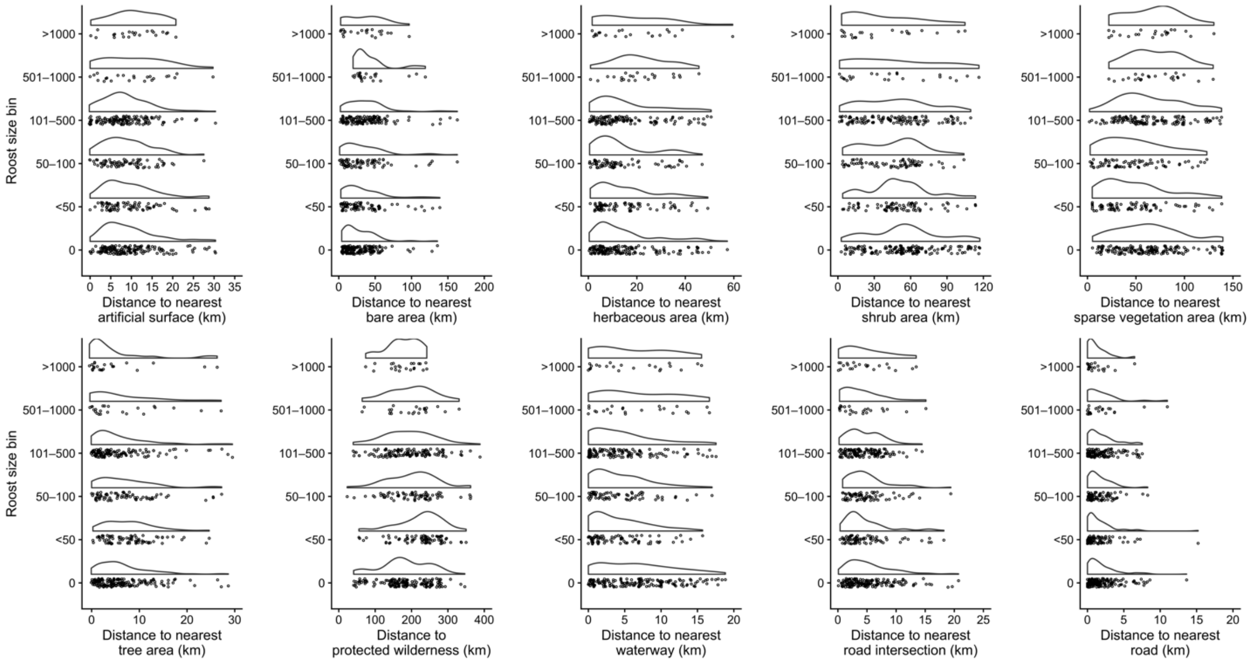

Figure S10. Distribution of roost sizes (including unoccupied roost sites) relative to land-use covariates. Raincloud plots show the statistical distribution of variables over individual points.

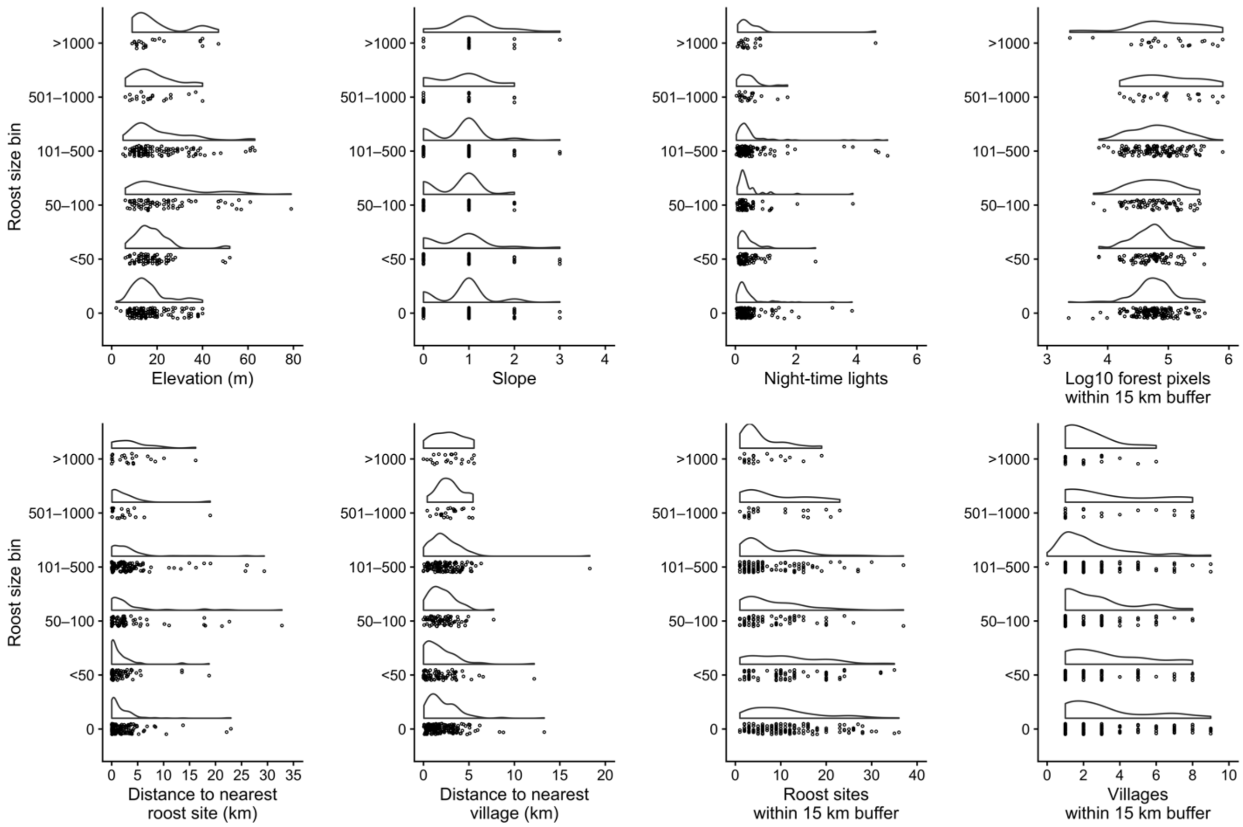

Figure S11. Distribution of roost sizes (including unoccupied roost sites) relative to remaining covariates. Raincloud plots show the statistical distribution of variables over individual points.

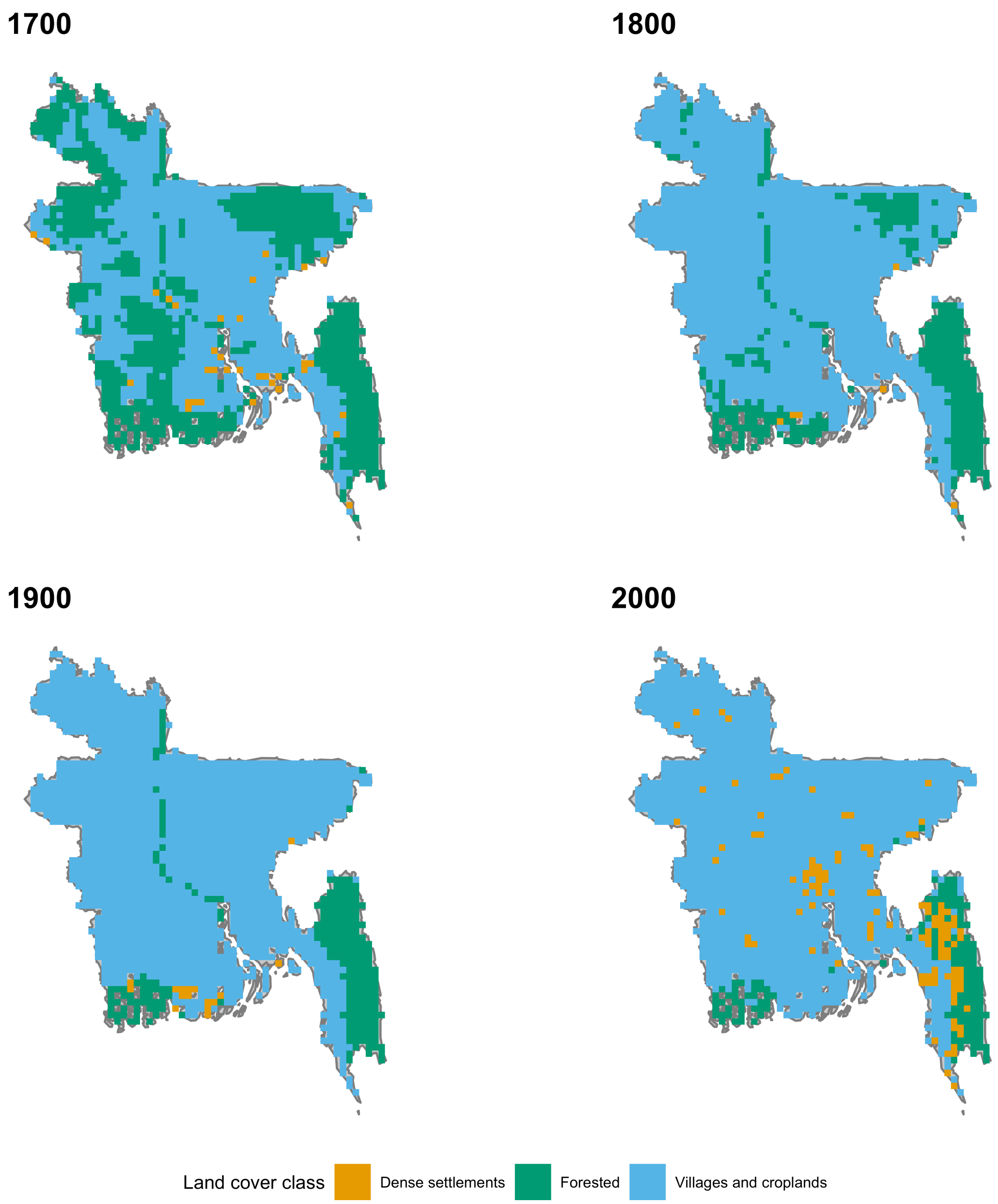

Figure S12. Maps of historical change in land cover across Bangladesh. Land cover classes were modified from data in Ellis et al. [6].

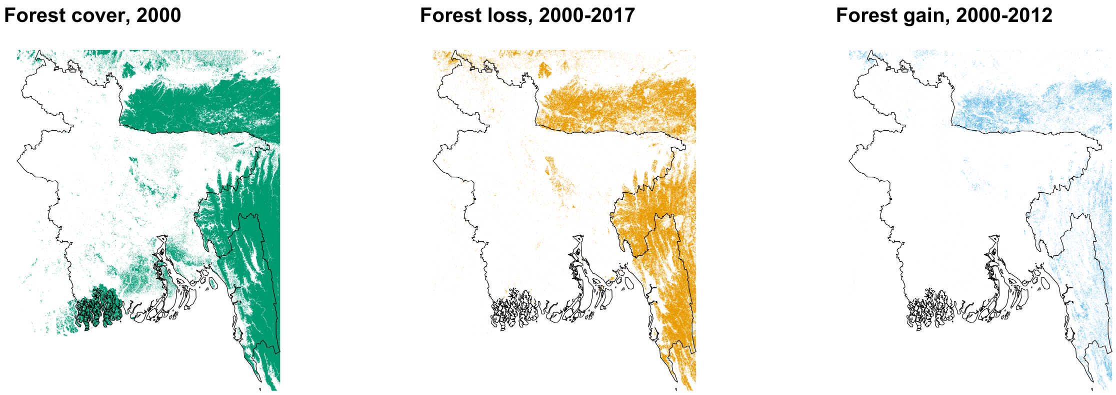

Figure S13. Maps of forest cover change in Bangladesh since 2000. Data were drawn from Hansen et al. [5]. Only pixels with forest cover >10% are shown while forest loss and gain within a pixel is binary. Note that forest cover gain only covers the period 2000–2012.

References

1. Fick, S.E.; Hijmans, R.J. WorldClim 2: new 1-km spatial resolution climate surfaces for global land areas. *Int. J. Climatol.* **2017**, *37*, 4302–4315, doi:10.1002/joc.5086.
2. Lloyd, C.T.; Sorichetta, A.; Tatem, A.J. High resolution global gridded data for use in population studies. *Sci. Data* **2017**, *4*, 170001, doi:10.1038/sdata.2017.1.
3. Lloyd, C.T.; Chamberlain, H.; Kerr, D.; Yetman, G.; Pistolesi, L.; Stevens, F.R.; Gaughan, A.E.; Nieves, J.J.; Hornby, G.; MacManus, K.; et al. Global spatio-temporally harmonised datasets for producing high-resolution gridded population distribution datasets. *Big Earth Data* **2019**, *3*, 108–139, doi:10.1080/20964471.2019.1625151.
4. Doxsey-Whitfield, E.; MacManus, K.; Adamo, S.B.; Pistolesi, L.; Squires, J.; Borkovska, O.; Baptista, S.R. Taking advantage of the improved availability of census data: a first look at the gridded population of the world, version 4. *Pap. Appl. Geogr.* **2015**, *1*, 226–234, doi:10.1080/23754931.2015.1014272.
5. Hansen, M.C.; Potapov, P. V; Moore, R.; Hancher, M.; Turubanova, S.A.; Tyukavina, A.; Thau, D.; Stehman, S. V; Goetz, S.J.; Loveland, T.R.; et al. High-resolution global maps of 21st-century forest cover change. *Science.* **2013**, *342*, 850–853, doi:10.1126/science.1244693.
6. Ellis, E.C.; Klein Goldewijk, K.; Siebert, S.; Lightman, D.; Ramankutty, N. Anthropogenic transformation of the biomes, 1700 to 2000. *Glob. Ecol. Biogeogr.* **2010**, *19*, 589–606, doi:10.1111/j.1466-8238.2010.00540.x.
